# Antimicrobial and anti-biofilm activity of silver nanoparticles biosynthesized with *Cystoseira* algae extracts

**DOI:** 10.1007/s00775-023-01999-y

**Published:** 2023-04-21

**Authors:** Mário Fernandes, Noelia González-Ballesteros, André da Costa, Raúl Machado, Andreia C. Gomes, Maria Carmen Rodríguez-Argüelles

**Affiliations:** 1 Centre of Molecular and Environmental Biology (CBMA)/Aquatic Research Network (ARNET) Associate Laboratory, Department of Biology, Universidade do Minho, Campus de Gualtar, 4710-057 Braga, Portugal; 2 Institute of Science and Innovation for Sustainability (IB-S), Universidade do Minho, Campus de Gualtar, 4710-057 Braga, Portugal; 3 Departamento de Química Inorgánica, Universidade de Vigo, 36310 Vigo, Spain

**Keywords:** Silver nanoparticles, Gold nanoparticles, Green synthesis, *Cystoseira* sp., Antimicrobial activity, Antibiofilm activity

## Abstract

Antimicrobial resistance is an ever-growing global concern to public health with no clear or immediate solution. Silver nanoparticles (AgNPs) have long been proposed as efficient agents to fight the growing number of antibiotic-resistant strains. However, the synthesis of these particles is often linked to high costs and the use of toxic, hazardous chemicals, with environmental and health impact. In this study, we successfully produced AgNPs by green synthesis with the aid of the extract of two brown algae—*Cystoseira baccata* (CB) and *Cystoseira tamariscifolia* (CT)—and characterized their physico-chemical properties. The NPs produced in both cases (Ag@CB and Ag@CT) present similar sizes, with mean diameters of around 22 nm. The antioxidant activity of the extracts and the NPs was evaluated, with the extracts showing important antioxidant activity. The bacteriostatic and bactericidal properties of both Ag@CB and Ag@CT were tested and compared with gold NPs produced in the same algae extracts as previously reported. AgNPs demonstrated the strongest bacteriostatic and bactericidal properties, at concentrations as low as 2.16 µg/mL against *Pseudomonas aeruginosa* and *Escherichia coli*. Finally, the capacity of these samples to prevent the formation of biofilms characteristic of infections with a poorer outcome was assessed, obtaining similar results. This work points towards an alternative for the treatment of bacterial infections, even biofilm-inducing, with the possibility of minimizing the risk of drug resistance, albeit the necessary caution implied using metallic NPs.

**Graphical abstract:**

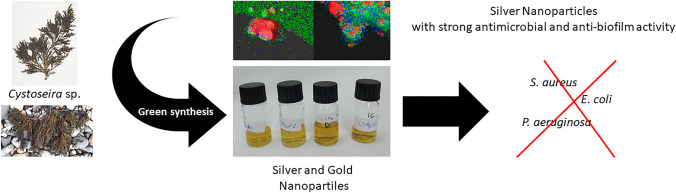

## Introduction

Bacterial infections remain as a major threat to human health as one of the ten leading causes of death worldwide. Although the development of antibiotics has helped to control bacterial infections and reduce the number of deaths, the misuse, over-use and long-term treatment with traditional antibiotics has increased the threat of antibiotic-resistance bacteria [1, 2]. This exponential increase of antibiotic-resistant bacteria calls for effective and more targeted therapies to treat those infections. This resistance can be acquired either by the accumulation of multiple genes, each responsible for the resistance of a single bioactive compound, or by the exponential expression of genes associated with multidrug efflux pumps that exclude the bioactive drugs from the bacteria [3].

In the search for alternative treatments against bacterial infections, nanotechnology has emerged as a remarkable strategy, thanks to the great variety of available nanomaterials that have potential antimicrobial activity. Some of the interest in this type of materials relies in their tunability and multifunctionality. Their physico-chemical properties, such as size, shape and surface chemistry, are easily controlled, increasing their potential applications. Also, they can possess intrinsic activity or act as a delivery vehicle for other active compounds [4, 5]. In this regard, silver nanoparticles (AgNPs) represent an interesting alternative as an antimicrobial agent against multiple pathogens [6, 7]. They stand out as some of the most promising nanomaterials to combat bacterial infections, due to their improved penetration inside microbial cells, reactive oxygen species (ROS) and free radical generation, and modulation of microbial signal transduction pathways [8].

Unfortunately, the application of nanoparticles (NPs) in medicine is still at its early stage. Great concern has been given to the toxicity of materials for its use in the pharmacological sector, which could be related with the use of toxic reagents and capping agents during synthesis. To overcome this drawback, researchers have moved towards greener routes of production of NPs using natural compounds with the aim to increase the biocompatibility and functionalize the NPs [8]. Among the different strategies employed, the use of macroalgae for the synthesis of NPs has attracted considerable attention [9].

Macroalgae represent a vast source of bioactive compounds with potential applications in different fields, for instance in medicine [10–13]. In particular, brown seaweeds possess natural compounds such as polysaccharides, phenols and terpenes, steroids, phlorotannins, and lipids known to possess strong antioxidant, anti-inflammatory, anti-viral, anti-tumor and anti-diabetes properties [14].

Previously, we synthesized gold nanoparticles (AuNPs) with aqueous extracts of brown seaweeds *C. baccata* and *C. tamariscifolia* and demonstrated their good, non-cytotoxic, bioactivity towards cell regeneration and anti-tumoral properties [15, 16]. In recent years, research on the antimicrobial potential of AuNPs has increased considerably, and findings show that these NPs trigger microbial cell damage as a result of oxidative stress, membrane and DNA damage. Among the advantages that they present, one can highlight their biosafety, the possibility of adapting their design to regulate gold nanomaterial excretion/metabolism, the potential of using different molecules to modify their surface and of enhancing antibacterial effects by manipulating size, shape, and surface properties [17, 18].

Here, we describe the synthesis and characterization of AgNPs produced with extracts of these algae and evaluated antimicrobial activity of both AgNPs and AuNPs against three bacterial species of clinical relevance known to develop a multidrug resistant phenotype: *Escherichia coli*, *Pseudomonas aeruginosa* and *Staphylococcus aureus* [19–21]**.**

## Materials and methods

### Preparation and characterization of algal extracts

Thalli of live bunches of *C. baccata* (CB) and *C. tamariscifolia* (CT) were collected at the lower intertidal rocky shore in the NW coast of Spain (42º12′2.9″N; 8º47′6.2″W) and in the NW coast of Portugal (N 41 47.858′ W 008 52.423′), respectively. The algal extracts were prepared as previously reported [15, 16].

### Synthesis of silver nanoparticles (Ag@CB and Ag@CT)

Optimal reaction conditions for the synthesis of AgNPs using *C. baccata* (Ag@CB) or *C. tamariscifolia* (Ag@CT) were determined after several trials with different ratios of seaweed extracts and silver nitrate salt, different temperatures, and time. Briefly, for the synthesis of Ag@CB, 50 mL of CB extract at a concentration of 4 × 10^4^ μg/mL was heated at 100 °C. Then, 2 mL of 0.005 M silver nitrate solution was slowly added to the extract; the solution was kept at the selected temperature, while stirring for 30 min. In the case of Ag@CT, 60 mL of CT extract (1.7 × 10^4^) was heated at 100 °C. Then, 2 mL of 0.005 M silver nitrate solution was slowly added to the extract; the solution was kept at the selected temperature, while stirring for 30 min. In all cases, the reaction was followed by UV–Vis spectroscopy.

### Characterization of Ag@CB and Ag@CT

A Jasco Spectrometer V-670 was used for the acquisition of UV–Vis spectra at room temperature. Zeta potential of Ag@CB and Ag@CT was obtained through electrophoretic mobility by taking the average of five measurements at the stationary level using a ZetasizerNano S (Malvern Instruments, Malvern U.K.) equipped with 4 mW He − Ne laser, operating at a wavelength of 633 nm. Samples for Fourier transform infrared spectroscopic analysis (FTIR) were prepared placing the extracts and the NPs solutions in an oven at 80 °C until dry. The dried materials were ground to fine powder and used to record the spectra in transmittance mode employing KBr pellet technique. FTIR spectra of the extracts and NPs were recorded using a Jasco FT/IR-6100 spectrophotometer in the range of 4000–400 cm^−1^ at a resolution of 4 cm^−1^.

Ag@CB and Ag@CT samples for electron microscopy characterization were centrifuged at 10,000 rpm for 30 min to eliminate part of the extract. Then, the pellets were dispersed in milliQ water and sonicated for 15 min. Finally, a drop of the NPs’ dispersions was placed onto holey carbon films supported on a copper grid. Transmission electron microscopy (TEM) images were acquired with a JEOL JEM 1010 (100 kV), while high-resolution transmission microscopy (HRTEM) and scanning transmission electron microscopy (STEM) images were acquired with a JEOL JEM 2010F or with a JEOL JEM 2200FS field emission gun TEM operated at 200 kV. Electron energy loss spectroscopy (EELS) measurements were performed in STEM mode using a Gatan Quantum EELS GIF, with a collection semi-angle of *β* = 16.75 mrad; the energy resolution was ∼ 1.75 eV (FWHM of the zero-loss peak). To avoid the contribution of the carbon film, EELS spectra were measured in areas of the sample positioned upon a hole. Coupling between the STEM unit and the EDS detector (Oxford Inca Energy 200) was used to obtain elemental maps. Data collection and analysis were carried out using Digital Micrograph software by Gatan.

### Antioxidant activity characterization

Three assays were performed to analyze the antioxidant and antiradical activity of Ag@CB and Ag@CT and results were compared with the data previously obtained for CB and CT extracts [16]. The DPPH radical scavenging activity, the reducing power and the total content of phenols were determined as earlier detailed [15, 22].

### Statistical analysis

GraphPad Prism 6 software was employed for the determination of significant differences between the antioxidant activity obtained for the different extracts before and after the synthesis of NPs, by performing of a one-way analysis of variance (either an ANOVA or a Kruskal–Wallis test) and a Tukey’s or Dunn’s test afterwards. All experiments were performed three times. In the graphs, results are expressed as: ns *P* > 0.05, *P ≤ 0.05, **P ≤ 0.01, ***P ≤ 0.001, ****P ≤ 0.0001.

### Antibacterial assays

Antibacterial assays were performed for silver and gold nanoparticles by determination of the minimum inhibitory concentration (MIC) and minimum bactericidal concentration (MBC) against a Gram-positive bacteria: *Staphylococcus aureus* ATCC 6538, and two Gram-negative bacteria: *Pseudomonas aeruginosa* ATCC 10,145 and *Escherichia coli* ATCC 11,303, following the European Committee on Antimicrobial Susceptibility Testing (EUCAST) and the Clinical Laboratory Standards Institute (CLSI) guidelines [2, 23]. Bacterial cell cultures were grown overnight at 37 °C in Mueller–Hinton Broth (MHB) and diluted to a final density of 1 × 10^6^ CFUs/mL. For the MIC assays, 50 μL of bacterial suspensions in MHB and 50 μL of serial diluted samples were mixed in 96-well plates and incubated overnight. The samples were tested at the following concentrations: CB extract between 140 and 1700 μg/mL; Ag@CB between 0.54 and 6.47 μg/mL [Ag]; Au@CB between 0.54 and 6.47 μg/mL [Au]; CT extract between 25 and 300 μg/mL; Ag@CT between 0.54 and 6.47 μg/mL [Ag]; Au@CT between 0.54 and 6.47 μg/mL [Au]; kanamycin and ampicillin between 5 and 60 μg/mL; and silver nitrate between 0.85 and 10.19 μg/mL. The MIC was determined as the lowest concentration with no visible growth by measuring the optical density at 600 nm. For determination of the MBC, 10 µL of each condition tested for MIC were diluted 1:100 (V:V) in saline solution 0.87%, and plated (50 µL) into Mueller–Hinton Agar (1.5% w/V). The plates were then incubated overnight at 37 °C and digitally recorded for colony forming units (CFUs) enumeration.

### Inhibition of biofilm formation

The inhibition of biofilm production was assessed in *S. aureus* ATCC 23,235 and *P. aeruginosa* PAO1, which have a mucoid phenotype and are capable of producing biofilms [24]. The cultures were grown overnight in MHB and diluted to a final density of 1 × 10^6^ CFUs/mL. Bacterial suspensions (50 μL) in MHB were mixed with samples solutions (50 μL) in 96-well plates and incubated at 37 °C for 18 h. The samples were tested at the same range of concentrations mentioned in the section above. Following incubation, the medium was removed, and the bacteria were fixed for 5 min with 200 µL methanol anhydrous 99.8%. The methanol solution was then removed, and cells stained with 200 µL of 0.2% crystal violet for 5 min, followed by 3 washing steps with PBS 1X. The crystals were solubilized with a solution of 33% (V/V) of acetic acid in PBS 1X and the absorbance was read at 570 nm.

### Live–dead assay

The live–dead assay was assessed in *E. coli* ATCC 11,303. Briefly, bacterial cells were grown overnight at 37 °C in Mueller–Hinton Broth (MHB). In the following day, 100 µL of bacterial culture diluted to a final density of 1 × 10^7^ CFUs/mL was incubated with AgNPs (Ag@CB and Ag@CT) at 0.54 μg/mL and 6.47 μg/mL overnight. All samples were then filtered through a 0.1 µm pore polycarbonate track-etched filter for fluorescence analysis (Sartorius) followed by filtration of 100 µL of a solution 1:1 (V/V) of SYTO™ 9/propidium iodide (PI). This filter was then observed in an Olympus BX63F2 fluorescence microscope with FITC and TRITC filters.

## Results

### Synthesis and characterization of Ag@CB and Ag@CT

In the present study, the potential application of the two brown seaweeds CB and CT for the synthesis of AgNPs was investigated. To attain homogeneous shape and narrow size distribution of AgNPs, several reaction conditions (extract concentration, AgNO_3_ concentration, temperature and time) were assayed and monitored by UV–Vis spectroscopy and TEM. First, synthesis was performed at room temperature; however, the reactions were too slow and NPs were not homogeneous. It was observed that increasing the temperature resulted in faster reactions and more homogenous shape and size of the particles. Similarly, it was observed that, when diluting the extracts, the synthesized NPs presented more homogeneous size and shape. Table 1 collects the final optimal reaction conditions.

**Table 1:** Optimal reaction conditions for silver nanoparticles synthesis

Algae	[Extract] (μg/mL)	[Ag] (μg/mL)	T (°C )	*t* (h)	Code
*C. baccata*	4 × 10^4^	27	100	0.5	Ag@CB
*C. tamariscifolia*	1.7 × 10^4^	21.6	100	0.5	Ag@CT

**Fig. 1:**
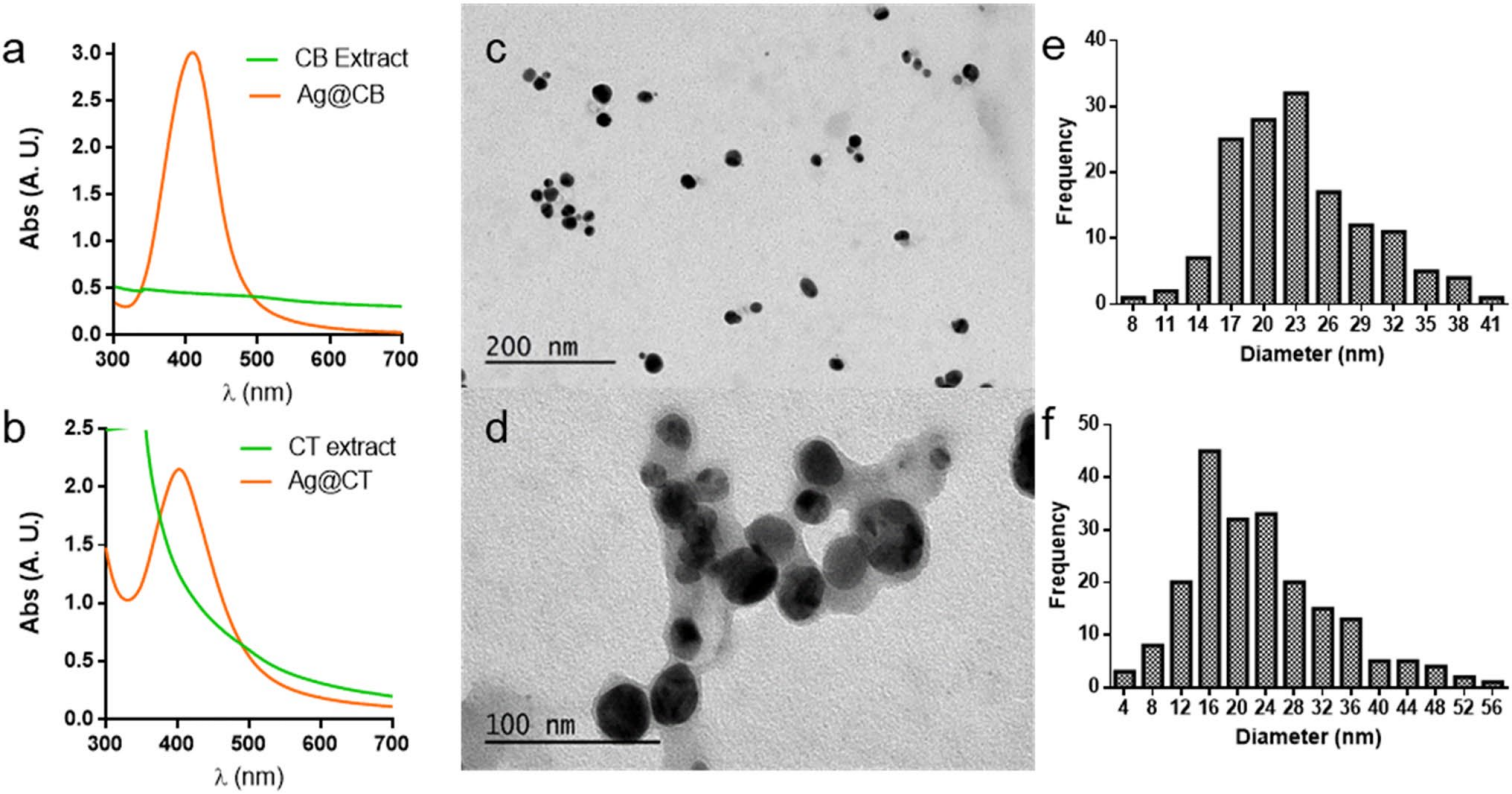
Ultraviolet–visible spectroscopic analysis of **a** Ag@CB and **b** Ag@CT. TEM images of **c** Ag@CB and **d** Ag@CT. Size distribution histogram of **e** Ag@CB and **f** Ag@CT

As previously indicated, the reactions were monitored by assessing color change and by UV–Vis spectroscopy. During the reaction, Ag(I) is reduced to Ag(0) and the characteristic surface plasmon resonance (SPR) band of AgNPs should appear at around 400 nm in contrast with the UV–Vis spectrum of the extract that do not absorb in this wavelength. For Ag@CB, an intense SPR band was observed (Fig. 1a), with *λ*_max_ at 410 nm after 30 min of reaction, while the SPR band of Ag@CT appears at 404 nm (Fig. 1b).

The stability and the surface charge of the AgNPs obtained were analyzed through measurement of the zeta potential. Values of − 34.3 ± 1.0 and − 26.9 ± 0.7 mV were obtained for Ag@CB and Ag@CT, respectively, indicating that the samples possess a negative surface charge. According to other reports, the high value obtain for both samples suggests high stability for the colloidal suspension which were proven to be stable for more than three months when preserved at 4 °C.

Size and shape of the synthesized NPs were characterized through transmission electron microscopy. As can be observed in the micrographs of Fig. 1c, d, the NPs synthesized with both seaweeds display a spherical morphology. In the case of Ag@CT, it can be clearly observed that a layer of organic matter is surrounding the NPs. Size distribution histograms were calculated after the measurement of at least 100 particles and are shown in Fig. 1e, f. It can be observed that the particles synthesized with the different seaweed present similar sizes, with mean diameters of 21.7 ± 6.2 nm and 22 ± 1.4 nm for Ag@CB and Ag@CT, respectively.

**Fig. 2:**
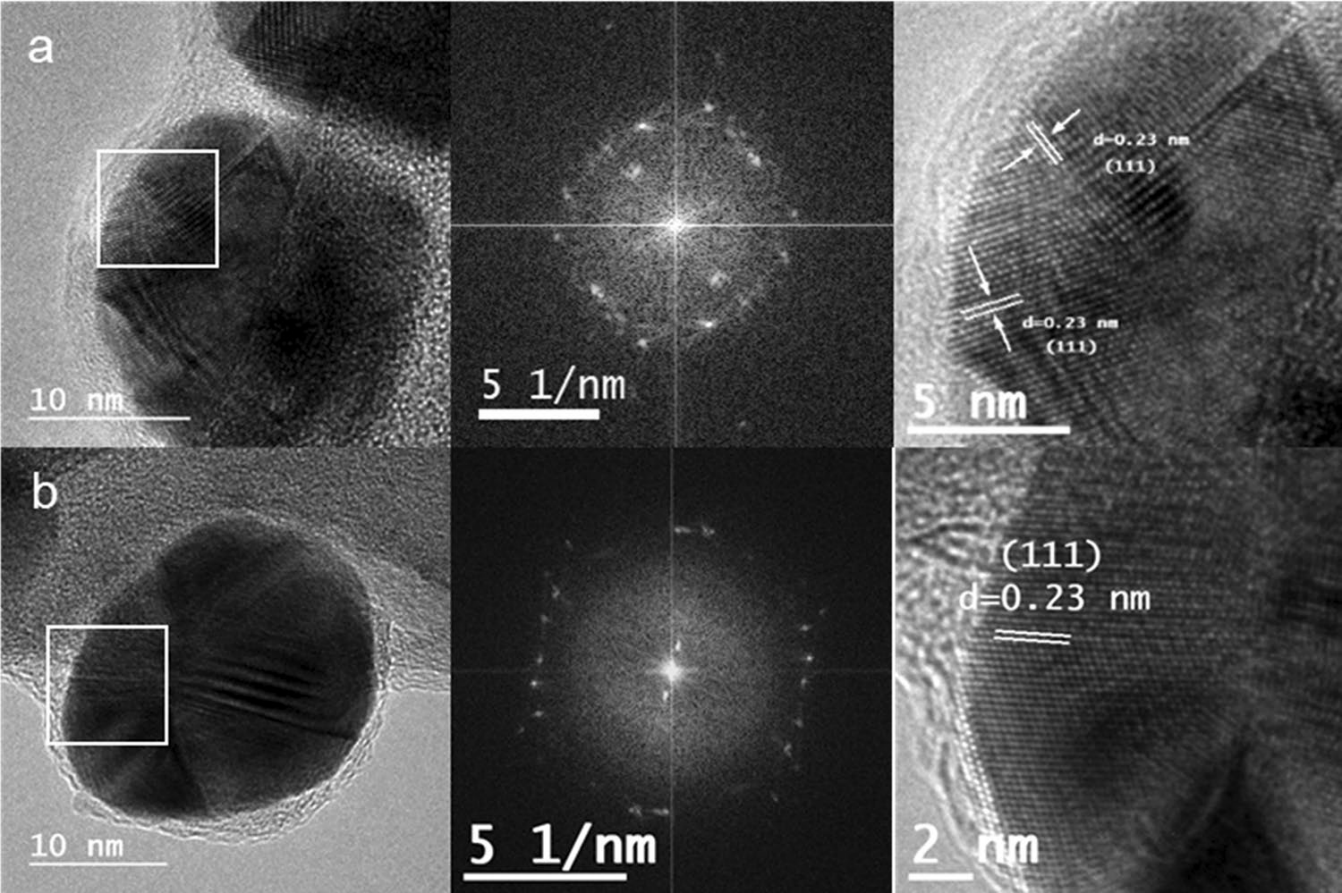
HRTEM images of **A** Ag@CB, **B** Ag@CT, with their corresponding Fourier transformed and the amplification of the selected area showing interplanar distance of the crystalline structure with the calculated d-spacing and their corresponding index Miller

HRTEM was also performed, and the images acquired are shown in Fig. 2 a and b together with the corresponding Fourier transform analysis. In both samples, it can be observed that the NPs display internal complex contrast, and the study of their Fourier transforms shows that they are polycrystalline. Furthermore, the interplanar distance of the crystalline structure was measured in the marked area of the selected NPs, followed by the assignation of the corresponding Miller index based on tabulated data. As shown in the Fig. 2, both samples presented the preferential d-spacing of 0.23 nm, corresponding to the Miller index (111) of face-centered cubic structure of silver.

**Fig. 3:**
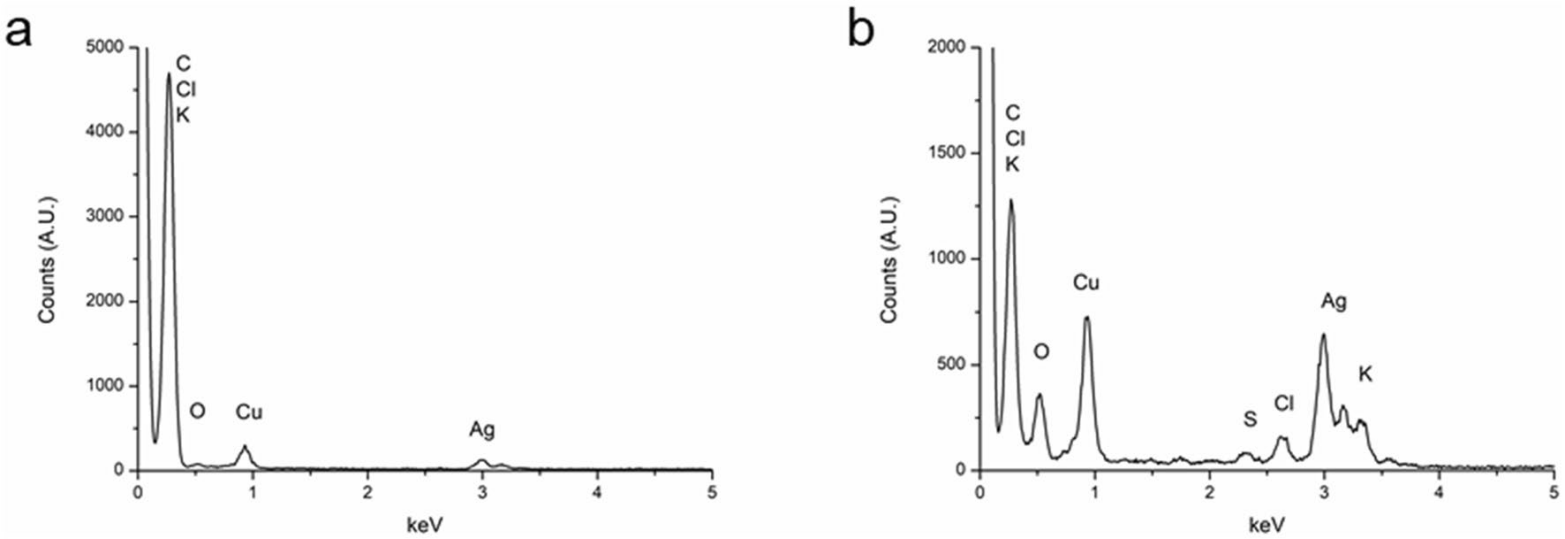
EDX spectra of **a** Ag@CB and **b** Ag@CT

**Fig. 4:**
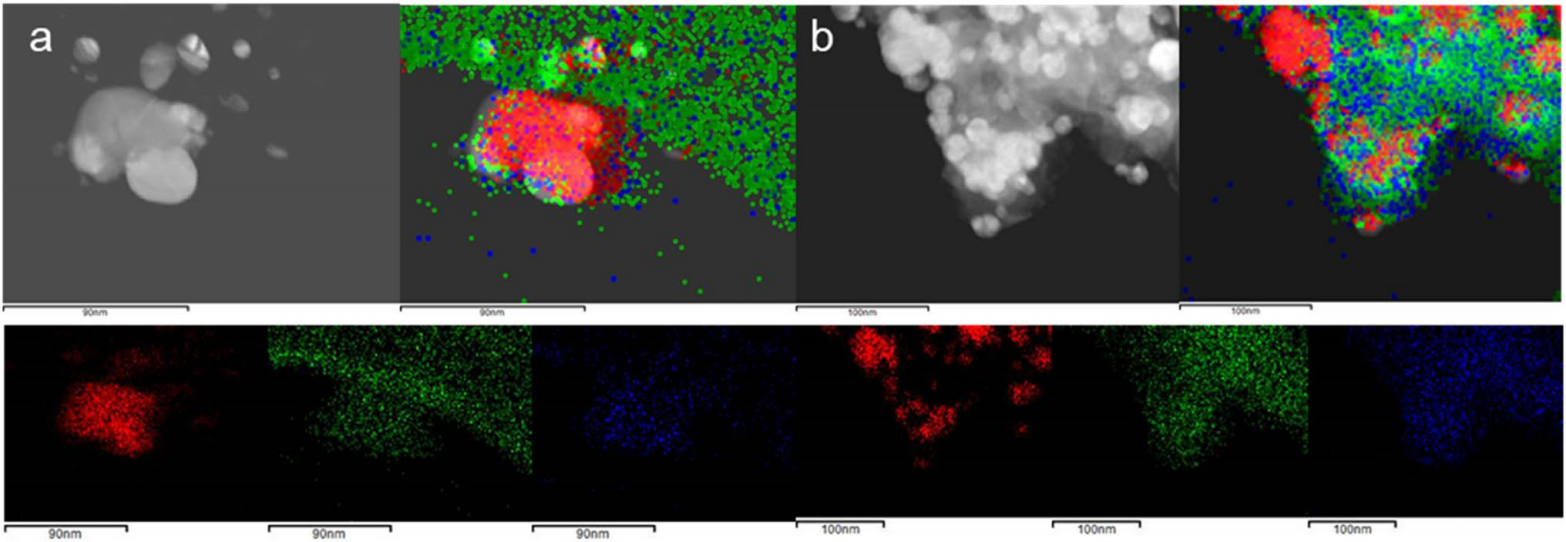
STEM images and elemental mappings [silver (red), carbon (green) and oxygen (blue)] of **a** Ag@CB and **b** Ag@CT

Energy-dispersive X-ray spectra were acquired (Fig. 3) in the areas shown in the STEM images (Fig. 4). In both spectra, apart from silver, appears the signal of other elements confirmed to be present in the seaweeds. In regards of Ag@CB, the spectrum showed the appearance of carbon, chorine, potassium and oxygen, while Ag@CT, apart from these elements, also show the presence of sulfur [25, 26]. It should be noted that the copper signal present in both cases could be due to the grids employed for samples preparation but can also be attributed to the composition of the seaweed, since there are studies confirming that copper can be accumulated in seaweeds [27].

From the EDX spectra, the elemental mapping of silver (red), carbon (green) and oxygen (blue) were obtained as well as a mix map. As can be observed in Fig. 4a, b, the elemental mapping shown a layer of carbon and oxygen around the NPs, while silver is concentrated in the NPs suggesting the full reduction of the metal salt employed.

FTIR spectra were obtained before and after the synthesis of Ag@CB and Ag@CT. For each seaweed, the assignation of the bands was made on the basis of our previous studies [15, 16] and other reports on the composition of *C. baccata* [28–30], *C. tamariscifolia* [25, 31] and other *Cystoseira* species [32–34].

**Fig. 5:**
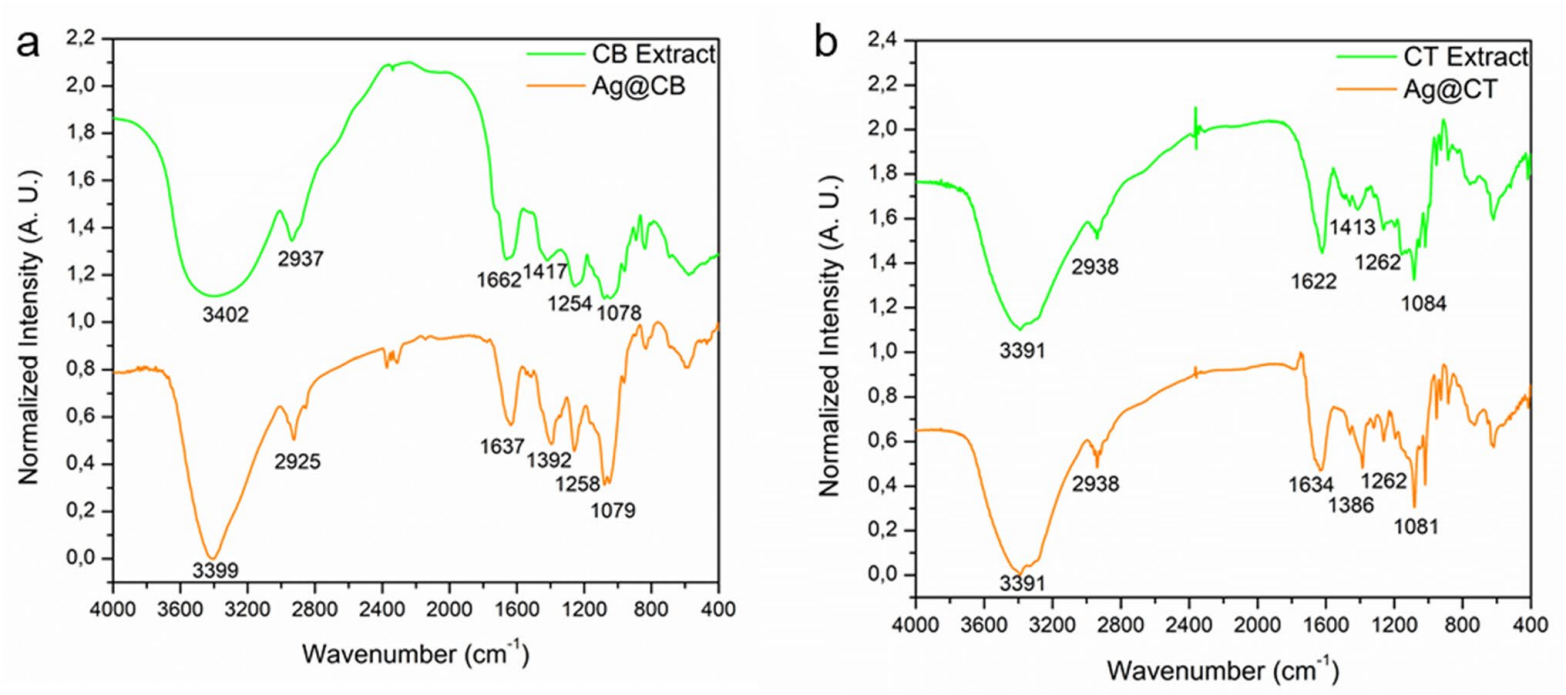
FTIR spectra of **a** CB extract [15] and Ag@CB and **b** CT extract [16] and Ag@CT

In summary, the infrared spectra shows the presence of different regions of interest. The first one between 4000 and 2000 cm^−1^ corresponds to the bands assigned to O–H (~ 3400 cm^−1^) and C–H (~ 2900 cm^−1^) stretching vibrations. In the second region, between 6000 and 4000 cm^−1^, appears the bands assigned to the carboxylate group of amides and to carbonyl groups. The next region, between 1200 and 800 cm^−1^, is common to all polysaccharides and the bands are assigned to C–C and C–O stretching and C–O–C and C–OH vibration. Finally, the presence of sulfur in the samples was confirmed by the bands at ~ 1250 and 800 cm^−1^ assigned to O=S=O asymmetric stretching C–O–S bending vibration (Fig. 5).

### In vitro antioxidant activity

**Fig. 6:**
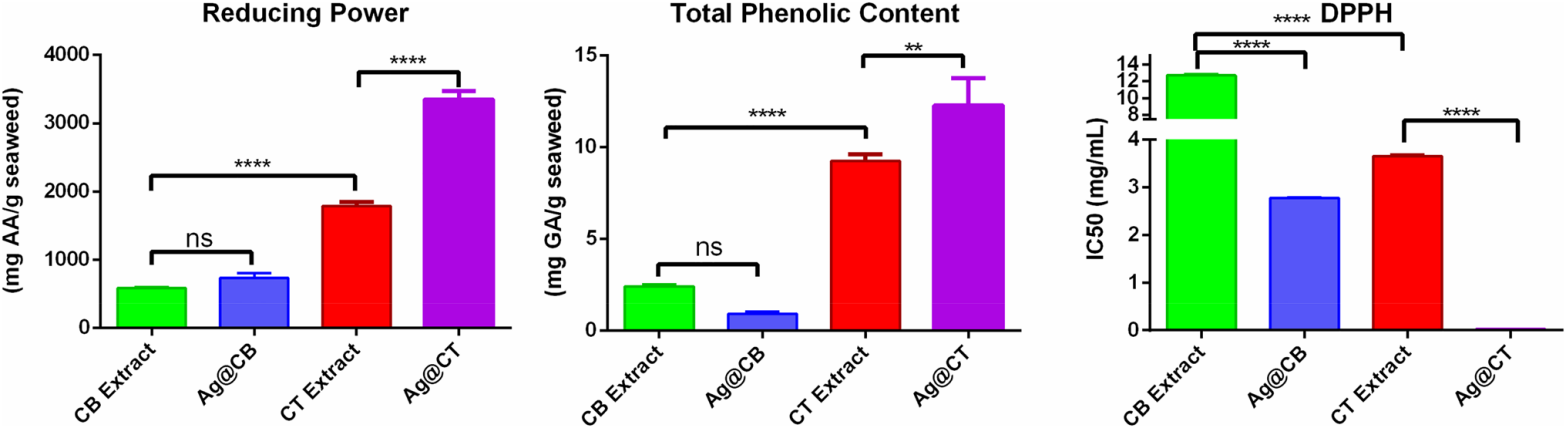
Reducing power, total phenolic content and DPPH scavenging activity of CB and CT extracts before [16] and after the synthesis of silver nanoparticles (Ag@CB and Ag@CT). In the graphs: ns *P* > 0.05, *P ≤ 0.05, **P ≤ 0.01, ***P ≤ 0.001, ****P ≤ 0.0001

In previous studies, we reported on the antioxidant activity of CB and CT extracts [15, 16]. In this work, the antioxidant activity of the extracts was compared with extracts containing the synthesized NPs. As it can be observed in Fig. 6, the CT extract presents much higher reducing power, total phenolic content, and DPPH scavenging activity than the CB extract. Interestingly, this difference between algae changes after the synthesis of Ag@CB and Ag@CT. First, in the case of CB, no significant difference can be observed in the reducing power, but a slight, not significant decrease in the total phenolic content is visible. However, a reduction in the IC50 value of DPPH indicates a higher scavenging activity in the presence of the NPs. On the other hand, in the case of CT extract, a significant increase in the reducing power, total phenolic content and DPPH scavenging activity are observed.

### Antibacterial activity

The antibacterial performance of the silver and gold nanoparticles, as well as the algae extracts, was assessed by determination of the MIC and MBC against three bacterial species of clinical relevance: *Escherichia coli* ATCC 11,303 (gram-negative), *Pseudomonas aeruginosa* ATCC 10,145 (gram-negative) and *Staphylococcus aureus* ATCC 6538 (gram-positive), in accordance with EUCAST/CLSI antimicrobial susceptibility recommendations. In all assays, three positive controls were used: silver nitrate, kanamycin—an aminoglycoside antibiotic, and ampicillin—a β-lactam antibiotic [35].

For the NPs produced with *C. tamariscifolia* (Ag@CT) and *C. baccata* (Ag@CB), it is evident that within the range of concentrations tested, all the bacteria are susceptible to the AgNPs (Table 2). In comparison, the AuNPs had only a modest effect.

**Table 2:** MIC and MBC concentrations (µg/mL) determined for CB and CT extracts, Ag@CB, Ag@CT, Au@CB, Au@CT and positive controls against *E. coli* ATCC 11,303*, P. aeruginosa* ATCC 10,145 and *S. aureus* ATCC 6538

	*E. coli*		*P. aeruginosa*		*S. aureus*	
	MIC (µg/mL)	MBC (µg/mL)	MIC (µg/mL)	MBC (µg/mL)	MIC (µg/mL)	MBC (µg/mL)
CB extract	> 1700	> 1700	> 1700	> 1700	> 1700	> 1700
CT extract	> 300	> 300	> 300	> 300	> 300	> 300
Ag@CB	4.31	4.31	2.16	6.47	4.31	4.31
Ag@CT	2.16	4.31	4.31	6.47	4.31	4.31
Au@CB	> 11.81	–	11.81	–	> 11.81	–
Au@CT	> 11.81	–	> 11.81	–	11.81	–
Ampicillin	40	60	60	60	60	60
Kanamycin	60	60	60	60	60	60
Silver Nitrate	10.19	10.19	10.19	10.19	6.79	10.19

The lowest MIC found for Ag@CT was obtained against *E. coli* (2.16 µg/mL) and against *P. aeruginosa* (2.16 µg/mL) for Ag@CB. The AgNPs were also able to cause a similar effect on *S. aureus*, although at a higher concentration (4.31 µg/mL). The AuNPs only reached measurable MIC for *P. aeruginosa* (11.81 µg/mL Au@CB) and *S. aureus* (11.81 µg/mL Au@CT). MBC was not attained with AuNPs against any of the microorganisms, as none of the tested concentrations completely inhibited colony formation after 24 h of exposure. Interestingly, the antimicrobial assays indicated that CT and CB extracts cause some growth inhibition, with CT at lower concentrations. Also, comparing with the silver nitrate control, both Ag@CT and Ag@CB demonstrated a higher antimicrobial efficacy, revealing lower MIC and MBC values (Table 2).

**Fig. 7:**
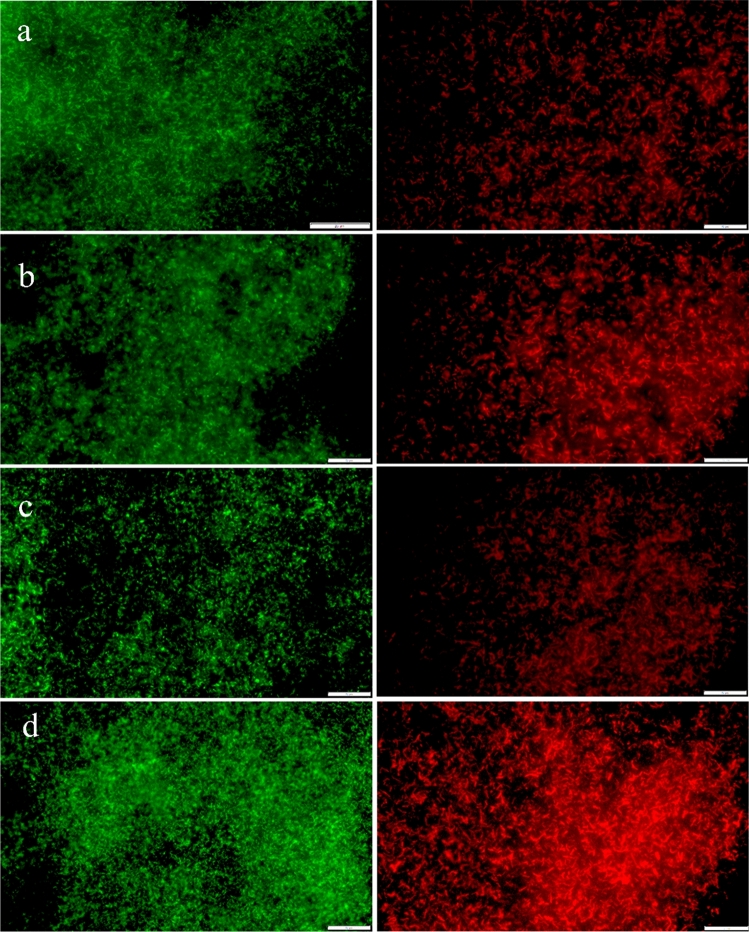
*E. coli* bacteria marked with SYTO™ 9 (green) and propidium iodide (red) fluorescent dyes: **a** Ag@CT 0.54 µg/mL, **b** Ag@CT 6.47 µg/mL, **c** Ag@CB 0.54 µg/mL, **d** Ag@CB 6.47 µg/mL. Scale: 20 µm

Remarkably, this antimicrobial effect was replicated in the live/dead assays regarding AgNPs. Representative experiments with *E. coli* (Fig. 7) illustrate that the concentration over MIC (6.47 µg/mL) was found to promote more cell death than in the concentration under the MIC (0.54 µg/mL). This was evidenced by an increase in the fluorescence of propidium iodide (PI), which marks cells undergoing late apoptosis or necrosis, evidenced by altered membrane permeability that allows this fluorescent compound to enter the cell. Bacteria incubated with Ag@CT showed approximately 69.5% increase in PI fluorescence intensity in concentrations over vs. under MIC, and Ag@CB induced approximately 76.3% increase in PI fluorescence intensity in concentrations over vs. below MIC.

The ability of the silver and gold nanoparticles produced with *Cystoseira* extracts to inhibit the formation of biofilms was evaluated using *P. aeruginosa* PAO1 and *S. aureus* ATCC 25,923 as model microorganisms [24]. The results obtained for the inhibition of production of these biofilms (Table 3) indicate that Ag@CT and Ag@CB have a MIC similar or lower to that of silver nitrate, except for one situation, and lower to the reference antibiotics ampicillin and kanamycin.

**Table 3:** MIC concentrations (μg/mL) determined for CB and CT extracts, Ag@CB, Ag@CT, Au@CB, Au@CT and positive controls against biofilm-producing bacteria *P. aeruginosa* PAO1 and *S. aureus* ATCC 25,923

	*P. aeruginosa* MIC (µg/mL)	*S. aureus* MIC (µg/mL)
CB extract	> 1700	> 1700
CT extract	> 300	> 300
Ag@CB	2.16	2.16
Ag@CT	4.31	4.31
Au@CB	11.81	11.81
Au@CT	11.81	11.81
Ampicillin	20	20
Kanamycin	20	20
Silver nitrate	4.31	2.16

We can observe that, for the range of concentrations tested, neither extract alone can exert an antimicrobial effect against the biofilm-producing bacteria. Both Ag@CT and Ag@CB demonstrated to effectively exert a strong antimicrobial effect at low concentrations. AuNPs, again, did show positive effects but at higher concentrations than with any AgNPs tested or the silver nitrate control. The best results were achieved with Ag@CB, with similar MIC (2.16 μg/mL for *S. aureus*) or lower MIC (2.16 μg/mL for *P. aeruginosa*) than silver nitrate control.

## Discussion

We successfully produced AgNPs with *C. baccata* and *C. tamariscifolia* extracts acting as reducing and stabilizing agents for the reaction. These NPs are polycrystalline and have a similar, small size—around 20 nm—when produced with the extract of both algae.

FTIR results prove the capping of the NPs with the extract resultant of the reduction of the polyphenols and other molecules. When comparing the CB extract with Ag@CB FTIR spectra (Fig. 5a), the shifts in bands may identify the main groups which might be responsible for the reduction of silver(I) and the capping of the NPs. It can be observed that the band at 3402 cm^−1^ shifted to lower wavelength (3392 cm^−1^) suggesting that the hydroxyl functional groups from polyphenols and polysaccharides might be involved in the reduction of Ag(I) ions to Ag(0). The band at 1662 cm^−1^ also shifted to lower wavelengths (1637 cm^−1^), suggesting that proteins are likely to cap NPs to prevent their agglomeration. In a similar way, the shift of the band from 1254 to 1258 cm^−1^ could indicate that sulfonic groups from polysaccharides are also involved in metal binding.

Regarding the CT Extract and Ag@CT, it can be noted that there are no changes in the band at 3391 cm^−1^. The major changes observed are associated with bands at 1622 cm^−1^ and 1413 cm^−1^ that shift to 1634 cm^−1^ and 1386 cm^−1^, respectively, indicating a strong participation of carbonyl groups in the reduction and stabilization of the NPs. Slight changes in the intensity and profile of the bands between 1200 and 1000 cm^−1^ can also be identified, suggesting the participation of polysaccharides in the mechanism.

The extracts of the two algae (especially CT) have good reducing power, total phenolic content, and DPPH scavenging activity. However, upon NP synthesis, Ag@CB display a higher scavenging activity, while Ag@CT associate with a significant increase in all three parameters. These results match the ones obtained in other studies regarding the enhancement of antioxidant activity of nanomaterials produced by green methods of synthesis [36–38]. A synergistic effect occurs, thanks to the presence of biomolecules with antioxidant activity capping the NPs. The high surface to volume ratio of small NPs increases the number of reactive sites, therefore increasing the desired property [39].

Ag@CT and Ag@CB revealed potent bacteriostatic and bactericidal effects against all the tested species, with quite low MIC and MBC values. In comparison, the bacteria were only modestly susceptible to the biogenic AuNPs. While the antimicrobial effect of silver is well known, the antimicrobial properties of gold are less studied and often regard the conjugation of AuNPs with antibiotics or drugs [40]. As for AgNPs, although the exact mechanism by which nanocrystalline silver causes bacterial cell death remains to be fully elucidated, it likely encompasses different modes of action mainly attributed to the release of silver ions (Ag^+^) [41, 42]. These include alterations of the bacterial cell wall and membrane, interaction with DNA, binding or inhibition of enzymes and membrane proteins, and increased level of reactive oxygen species (ROS) [42, 43]. AgNPs are able to penetrate the bacterial cell wall, attaching to cell membrane and causing structural changes and permeability, and altering transport activity [41, 43, 44]. In addition, AgNPs release silver ions (Ag^+^), which are the main active component of AgNPs, causing additional damage to bacterial cell membranes, accumulating inside the cell and affecting several vital functions [41, 43, 44]. Previously, Bondarenko et al., demonstrated that the antimicrobial action of AgNPs depends exclusively on the effective concentration of Ag^+^ inside the bacterial cells, deriving from the dissolution of AgNPs [41]. This multilevel conjugated action of AgNPs and silver ions (the effective active component), ultimately results in cell death. Here, the live/dead assays demonstrated obvious cell membrane permeabilization at concentrations below MIC and MBC. This suggests that Ag@CT and Ag@CB alter membrane permeability before an effective antibacterial activity takes place (either by inhibiting growth or killing cells). AgNPs are able to physically interact with the cell surface of bacteria, causing structural changes, and subsequently, affect its permeability [41, 43, 44]. Therefore, a cell may have its metabolic activity compromised before disruption of the cell envelope and subsequent cell lysis [45].

While described for its multidrug phenotype strains, with the presence of different efflux pumps [46], Gram-positive bacteria also have a cell wall that is permeable to most compounds and rarely restricts the internalization of antimicrobials [47]. Gram-negative bacteria, however, have an outer membrane external to their peptidoglycan cell wall that can work as a barrier [48]. Previously, Alzahrani et al. produced AgNPs with size between 60 and 114 nm from methanol extracts of *Galaxaura rugosa* and tested against several drug-resistant bacteria, with MIC values of 563 μg/mL for *E. coli* and *P. aeruginosa*, and 1500 μg/mL for *S. aureus* [49]. Ulagesan et al*.* reported similar results with AgNPs of 20–22 nm produced with *Pyropia yezoensis* extract, with MIC of 200 μg/mL and MBC of 400 μg/mL for *P. aeruginosa* [50]. Remarkably, the AgNPs produced with *Cystoseira* extracts in our study demonstrated to be comparatively more potent (Ag@CT with MIC values of 2.16 μg/mL for *E. coli* and 4.31 μg/mL for *P. aeruginosa* and *S. aureus*). This difference in the antibacterial potency is probably explained to some extent by the different size of the NPs and the capping biomolecules derived from the *Cystoseira* extracts. The antibacterial activity of AgNPs is largely mediated by the release and accumulation of silver ions. Therefore, changes in size are likely associated with the release kinetics of Ag^+^, with smaller NPs releasing a higher amount of silver ions, due to the large surface area; therefore, capping agents can alter the dissolution behavior, interfering with the release of silver ions [44]. For instance, Martínez-Castañón et al*.* demonstrated that reducing the size of AgNPs can improve their antibacterial properties [51]. Also, Morones et al., studied the effect of NP size in the bactericidal performance against gram-negative bacteria, demonstrating a size-dependent antibacterial activity [52]. Interestingly, the antimicrobial assays indicated that both CT and CB extracts inhibit the growth of the gram-positive bacteria *S. aureus* to some extent. As a gram-positive bacteria, this may be explained by the permeability of its cell wall, to most compounds, even antimicrobials [47]. Both Ag@CT and Ag@CB NPs demonstrated a higher antimicrobial efficacy compared to the silver nitrate control, with lower MIC and MBC values. Together with the recognized cytotoxicity of the silver nitrate to human cells at these concentrations [53], the better performance of the AgNPs is strengthened.

During infection, bacteria may organize into biofilms as protection from harmful conditions [54]. So, for a long-term bactericidal treatment, the effectiveness of a determined compound can diminish if it fails to penetrate the biofilm produced by the bacteria. These phenomena are becoming more common in recent years, turning into a serious health issue especially in developing countries [55]. AgNPs offer advantages for the treatment of biofilms as AgNPs and Ag^+^ ions can penetrate through the extracellular components and interact with the multiple components of biofilms, interfering with bacterial metabolism and affecting vital functions [44]. However, the size of AgNPs is of paramount importance as biofilm penetration can be obstructed for particles larger than 50 nm [44]. In this study, at concentrations of 2.16 µg/mL the biogenic AgNPs produced with *Cystoseira* showed a strong inhibitory effect against biofilm-producing bacteria, with better results than silver itself, and certainly more than the algal extract alone. Öztürk et al*.* found that AgNPs produced with the algae *Gelidium corneum* extract could inhibit the growth of biofilm by 50% at 50 μg/mL, which are inhibition values very close to those of Ag@CB and Ag@CT [56]. Similarly, Danaei et al*.* found that AgNPs produced with *Spirogyra sp.* had some success in inhibiting the formation of biofilms by this bacteria [57]. On the other hand, AgNPs using *Oscillatoria sp.* extract showed poor capacity to inhibit the growth of the biofilms formed by *P. aeruginosa*, thus never reaching a MIC [58]. Interestingly, most mechanistic information regarding this putative application for biogenic AgNPs stems from studies with plant extracts. As an example, in Mohanta et al*.*, AgNPs produced with extracts of Indian medicinal plants *G. lanceolarium*, *S. anacardium*, and *B. retusa* showed anti-biofilm activity against *P. aeruginosa* but with generally higher MICs (68.94 ± 0.2 μg/mL, 12.9 ± 0.2 μg/mL, and 23.48 ± 0.2 μg/mL, respectively) [59]. The results obtained with *E. coli* and *S. aureus* were slightly poorer. In combination with other studies, the authors suggest a mechanism involving biosorption and Ag^+^ ions release from the NPs that penetrate into the biofilm [60]. This may interfere with synthesis and secretion of exopolysaccharides (EPSs) and kill bacteria [59]. As mentioned, size, shape and surface characteristics influence interaction of NPs with biofilms, with size being a major player [61]. As we obtained very small AgNPs (average diameter < 25 nm), they fit into the ideal range for biofilm-infection control (5-100 nm), favoring biofilm penetration [60, 62]. This argument may explain the low MIC values obtained in our study, significantly lower than most other reports in the literature, mentioned earlier, which tested NPs with approximately 100 nm in diameter. Despite the efficient antibacterial effect of AgNPs, one should take into consideration that the potential harmful effects of AgNPs are subject of intense debate [42, 63, 64]. Nevertheless, studies specifically addressing the correlation between AgNP physico-chemical properties and cytotoxicity mechanisms, both against human cells and microorganisms, particularly biofilm-producing bacteria, are still limited.

From this study, *Cystoseira*-produced AgNPs emerge as an interesting alternative for the safe treatment of infections caused by both planktonic and sessile (biofilm-producing) bacteria.

## Data Availability

Data will be made available upon reasonable request.
